# PERCEIVED NEIGHBORHOOD CHARACTERISTICS AND COGNITIVE AGING: FINDINGS FROM THE SEATTLE LONGITUDINAL STUDY

**DOI:** 10.1093/geroni/igad104.1582

**Published:** 2023-12-21

**Authors:** Gizem Hueluer, Sherry Willis, Warner Schaie, Denis Gerstorf

**Affiliations:** University of Bonn, Bonn, Nordrhein-Westfalen, Germany; University of Washington, Seattle, Washington, United States; University of Washington, Seattle, Washington, United States; Humboldt University of Berlin, Berlin, Berlin, Germany

## Abstract

Lifespan developmental perspectives widely acknowledge that cognitive development and aging is shaped by an individual’s social, historical, and geographic context. The residential neighborhood is the most immediate geographic context of an individual. In this study, we used within-person longitudinal data from the Seattle Longitudinal Study to examine how cognitive aging trajectories in five primary mental abilities are associated with perceived characteristics of one’s residential neighborhood. 3,482 participants (aged 19 thru 100 years, 54% women) contributed a total of 8,021 observations over up to 35 years. Longitudinal trajectories were examined across age and analyses additionally controlled for sex, education, and income. Our findings showed that cleanness of air and availability of trees and open spaces were indeed associated with higher levels of most primary mental abilities at age 60. In addition, cleanness of air was associated with less pronounced cognitive aging for two of the five primary mental abilities and availability of trees and open spaces was associated with less pronounced cognitive aging for four of the five primary mental abilities. In contrast, noise, closeness to transportation, and feelings of safety showed only few associations with trajectories of cognitive aging, whereas familiarity with neighbors was associated with lower levels of most primary mental abilities at age 60. Taken together, the findings of this study demonstrate that characteristics of an individual’s neighborhood are related to trajectories of performance-based measures of cognitive aging over and above indicators of socio-economic status. We will discuss potential mechanisms underlying these associations and implications for future research.

